# Dynamic Structures of Bioactive Proteins as Determined by Nuclear Magnetic Resonance

**DOI:** 10.3390/ijms25010295

**Published:** 2023-12-25

**Authors:** Orsolya Toke, Gyula Batta

**Affiliations:** 1 NMR Research Laboratory, Centre for Structural Science, Research Centre for Natural Sciences, 2 Magyar tudósok körútja, H-1117 Budapest, Hungary; 2 Department of Organic Chemistry, Faculty of Science and Technology, University of Debrecen, Egyetem tér 1, H-4032 Debrecen, Hungary

According to “Panta rhei”, a phrase by the ancient Greeks, you cannot enter the same river two times. Everything changes, and this is true for biomolecules as well. NMR (nuclear magnetic resonance) spectroscopy provides both structural and dynamic information on molecules at atomic resolution, helping to understand the details of molecular recognition in protein interactions and offering a mechanistic insight into biochemical function and misfunction in cellular processes [1,2]. The ongoing progress in the development of NMR methods boosted with stable isotope labelling (^15^N, ^13^C, ^2^H, ^19^F) has significantly improved the limits of available protein size both in solution and in solid state, allowing the investigation of complex biological systems in near-physiological environments or even in the cell [3]. One of the advantages of NMR over current structural methods is that it can sample intramolecular and global motions in a broad time range of nanoseconds to seconds. Hence, the dynamics in folded proteins and even in disordered proteins (IDPs) can be monitored with high resolution and sensitivity. Importantly, NMR has become a powerful tool for elucidating protein dynamics involving transiently formed conformational states, which play an important role in binding, folding, and stability [4]. The capability of NMR to relate atomic-level structural and dynamic information to macroscopic kinetic and thermodynamic parameters makes it a unique and indispensable tool in biomedical research. The intention of this Special Issue was to highlight the current capabilities of NMR in deciphering the mechanism of action of proteins, and thereby improve our understanding of the differences between physiological and pathological states, with the aim of stimulating the development of new therapies.

Protein–protein interactions play a crucial role in biological processes. The modulation of these interactions by specific molecules is a promising therapeutic approach to combating disease states. NMR spectroscopy is a powerful tool to investigate binding affinity, interaction surfaces, and binding-induced conformational changes. Moreover, it has the ability to address these questions from the point of view of both the interacting proteins as well as the small molecules designed to interfere with the protein–protein interaction. The paper by Pagano et al. [5] focuses on the inhibition of the fibroblast growth factor 2 (FGF2)/fibroblast growth factor receptor (FGFR) signaling pathway [6], whose aberrant activation has a role in cancer development, vascular diseases, and viral infections. To gain insight into the inhibitory mechanism of selected polyphenolic molecules, NMR diffusion measurements (DOSY) and chemical shift perturbation mapping were employed. Specifically, following the determination of the effect of potential inhibitors on the equilibrium between free and complexed protein species using DOSY, the authors correlate these findings with residue-level analysis of the interaction mode, unveiling the different modes of action of the investigated polyphenolic molecules.

Understanding protein–nucleotide interactions with or without Mg^2+^ ions is the main goal of the study by Gadanecz et al. [7]. Their work is directed at catalytically significant states of the oncogenic G12C variant of KRAS, a ‘molecular switch’ located in the cell membrane regulating cell growth, proliferation, and survival [8]. The nucleotide-bound Mg^2+^-free state of GDP-loaded KRAS has previously been found to have a key role in the KRAS cycle, preparing KRAS for an interaction with the incoming nucleotide exchange factor (GEF) and subsequent reactivation, making it a promising target in therapeutic strategies [9]. Accordingly, Mg^2+^-free forms of oncogenic mutants might reveal structural differences, which would allow for the mutation-specific targeting of nucleotide exchange in KRAS. To overcome the difficulty posed by the high flexibility of the catalytically important Switch-I and Switch-II regions in Mg^2+^-bound and Mg^2+^-free G12C KRAS:GDP, resulting in a lack of distance restraints, the authors turned to Chemical-Shift-Rosetta (CS-Rosetta), a set of computational methodology tools allowing for protein structure determination based on backbone chemical shifts. To include the nonprotein components of the complex, they developed a novel protocol of extracting torsional angle distributions based on the CS-Rosetta ensembles, which were subsequently used as a restraint set for MD simulations. Overriding the force-field-based parametrization in the presence of the reinserted cofactors allowed for a detailed comparison of the Mg^2+^-bound and Mg^2+^-free states of G12C KRAS:GDP, providing new structural and dynamic insights into its role in the KRAS cycle. A detailed description of the Rosetta approach for protein structure determination, including some of the new developments such as incorporating data from hydrogen–deuterium exchange and paramagnetic NMR measurements, is given in a review by Koehler Leman and Künze [10].

As exemplified by KRAS, proteins are inherently dynamic in nature, and there is an intimate relationship between flexibility and function. Protein dynamics contributes to the thermodynamic stability of functional states and plays an important role in ligand binding, catalysis, and allostery. The work by Tollinger and coworkers [11] uses NMR relaxation dispersion (RD) techniques [12], a methodology suitable for inferring kinetic, thermodynamic, and structural information on sparsely populated, higher-energy invisible states in proteins, with implications for their function. The authors of [11] focus on a family of pathogenesis-related (PR) proteins, PR-10 [13], which represent a major source of allergic reactions to food. They use backbone amide ^15^N NMR relaxation dispersion spectroscopy to analyze the millisecond-timescale internal flexibility of thirteen PR-10 proteins from plant food sources in a comparative manner. While all of the allergens in their study possess an inherently flexible protein backbone in solution, protein-specific differences in the extent of structural flexibility are uncovered. A detailed analysis of PR-10 from peanut reveals the presence of at least two distinct conformational exchange processes on a millisecond-timescale involving two subglobal clusters of residues. In their study, the authors complement the ^15^N data with ligand-observed ^1^H relaxation dispersion measurements in the presence of the protein and show the dependence of RD profiles on the saturation level, which is a strong indication of ligand binding/release occurring on a millisecond time scale. Based on the chemical shift differences between the ground and the higher energy states inferred from the analysis, the authors hypothesize that the observed conformational transitions in PR-10 are related to ligand release from the internal cavity of the protein.

Conformational exchange between a closed and a more open state regulates ligand entry/release in intracellular lipid-binding proteins [14], as well, one of which is discussed in the Special Issue. Human ileal bile acid-binding protein (I-BABP) [15] has a key role in the enterohepatic circulation of bile salts [16], and its misfunctioning has been associated with a wide range of metabolic disorders and diseases of the gastrointestinal tract, including type 2 diabetes and colorectal cancer. The study presented herein [17] addresses the structural and dynamic determinants of the positive binding cooperativity [18] and site-selectivity [19] of bile salts with different hydroxylation patterns. As shown by the investigation of functionally impaired mutant human I-BABPs, conformational perturbations introduced in two pliable turn-regions are propagated to distant regions in the protein. Specific protein segments are identified, whose dynamic behavior shows subtle but consistent changes, with an observed alteration in positive cooperativity and site-selectivity in the defective variants. As suggested by the authors, the observed redistribution of motional freedom in the impaired variants raises the possibility of modifying the binding properties of human I-BABP by modulating the flexibility of specific protein regions in the protein.

Protein–ligand interactions and ligand specificity are also at the center of the study by Mineev and coworkers [20]. Their investigation is focused on NanoFAST, a member of the family of fluorogen-activating proteins (FAPs) [21,22], which bind small molecules that themselves are dim fluorescent agents but become active upon FAP binding. NanoFAST, a truncated variant of FAST that lacks 30 amino acid residues at its N-terminus [23], works exclusively with one out of all the known FAST ligands. To gain insight into the reasons for its specificity, a structural and dynamic NMR analysis of nanoFAST was performed in the *apo* and *holo* states. A comparison with the full-length form led to the conclusion that in the *apo* form of full-length FAST, the presence of the N-terminal domain destabilizes the dynamics of the C-terminal ‘core’ of the protein compared to nanoFAST. According to the authors, by acting as a free energy reservoir, enhanced µs-ms motion in the N-terminal domain makes ligand binding more favorable, leading to promiscuity in the fluorogen recognition of full-length FAST. On the contrary, the truncated analogue lacking a dynamic N-terminal segment selects a ligand with the most favorable packing mode, which results in highly specific ligand binding and activation.

Understanding not only how the amino acid sequence of a protein encodes the final native structure, but also the mechanism by which that structure is achieved, is one of the most difficult problems in biophysical chemistry. Folding/unfolding experiments performed in vitro yield information concerning protein folding mechanisms. To this end, several perturbation methods can be used, such as the addition of chemical denaturants, pH changes, and heat or cold denaturation. Czajlik et al. [24] use dimethyl-sulfoxide (DMSO)-induced chemical unfolding to explore how low-population states might influence the biological function of the antifungal protein PAF [25] and its inactive variant, PAF^D19S^. Due to the presence of multiple disulfide bonds, PAFs have highly stable folds around room temperature, but unfolded states are known to persist and contribute to thermal equilibrium in aqueous solution [26]. Unfolding was monitored by differential scanning calorimetry (DSC) and ^15^N-^1^H HSQC 2D NMR spectra. Additional NMR relaxation analysis was carried out for the two variants in pure aqueous solution and in 50 *v*/*v* % DMSO to determine the factors of thermodynamic stability of the active and inactive PAF variants.

The work by Dubois et al. [27] uses the alternative approach of high hydrostatic pressure to gain insight into the folding pathway of two small globular proteins. An advantage of pressure-induced unfolding [28,29] is the reversibility of the process (high pressure disfavors intermolecular protein interactions, thus preventing irreversible aggregation), allowing for the measurement of thermodynamic parameters for the folding/unfolding reaction. This allowed the authors to use 2D NMR spectroscopy to obtain residue-specific structural information along the folding reaction coordinate. The NMR-derived structural parameters were used to constrain distance geometry calculations [30] to obtain a topological description of the folding process of AVR-Pia and AVR-Pib, two ~90-residue proteins belonging to the MAX effector superfamily. The approach developed by the authors is suitable for obtaining atomic-level information on the full ensemble of conformers populated between 1 bar (native state) and 2500 bar (unfolded state) within a reasonable calculation time.

Despite methodological advances in the past decades, obtaining high-resolution structural and dynamic information on poorly crystallizable, poorly soluble molecular systems remains a challenge. As a barrier between the interior of the cell and the outside environment, the cell membrane and its proteinaceous part are important targets of therapeutic interventions. Solid-state NMR (ss-NMR) spectroscopy [31,32] is a powerful tool to obtain a mechanistic understanding of protein–membrane interactions. One approach relies on the derivation of orientational constraints for bonds and chemical groups from spectral parameters observed in samples of peptides/proteins embedded in lipid bilayers oriented between glass plates [33]. Ulrich and coworkers [34] combine this methodology with ^19^F-labeling to explore the possibility of manipulating the lateral pressure profile [35] of lipid bilayers, a physical parameter affecting the conformation and function of membrane proteins, using ‘crowder’ peptides residing in different regions of the bilayer. Specifically, by incorporating a ^19^F-labeled phenylglycine derivative in the middle of PGLa (a well-characterized alpha-helical antimicrobial peptide from *Xenopus laevis*), the orientation of PGLa with respect to the lipid bilayer, indicative of the degree of insertion, is monitored in the presence of various ‘crowders’ (e.g., surface-lying, transmembrane, tilted). As revealed by the ^19^F NMR measurements, upon the modulation of the lateral pressure profile of the membrane by the ‘crowder’ molecules, the depth of insertion of PGLa changes markedly. For instance, upon being exposed to transmembrane dimeric gramicidin A, PGLa stays in a surface-lying S-state, whereas in the presence of surface-bound magainin 2, PGLa inserts perpendicularly into the bilayer. As pointed out by the authors, peptides, with their broad spectrum of membrane interactions and location in the membrane, provide a more versatile tool for manipulating the lateral pressure profile of membranes than lipids themselves. This could have implications for modulating the function of ion channels and receptors, as well as the development of liposome-based drug delivery systems, with improved efficacy and reduced side effects. Likewise, as constituents of biomembranes, membrane proteins themselves may act as ‘crowders’ and have a significant effect (i) on the conformation and function of companion proteins, and (ii) on the binding of membrane-targeting agents, which opens new perspective for developing a more complex understanding of protein function in the membrane environment.

Another approach in solid-state NMR for obtaining atomic-level structural information is dipolar recoupling [36] under magic-angle spinning (MAS) [37,38], allowing for distance measurements between spin pairs at high precision (~0.1 Å). During spinning, the spatial orientations of the vectors connecting the spins are constantly changing, averaging out the orientation-dependent spin interactions responsible for unwanted spectral broadening. As structurally invaluable distance-dependent dipolar couplings are averaged out as well, dipolar recoupling techniques have been developed to selectively reintroduce dipolar couplings during MAS. A review paper [39] in this Special Issue is dedicated to rotational echo double resonance (REDOR) [40,41], a robust and versatile ss-NMR spectroscopic tool with the capability of providing intra- and intermolecular distances in multicomponent, heterogeneous systems. REDOR is used extensively as a spectroscopic ruler between isolated spins (e.g., ^13^C–^15^N, ^13^C–^31^P, ^13^C–^19^F) in site-specifically labeled systems, and more recently as a building block in multidimensional ss-NMR pulse sequences. This allows the simultaneous measurement of multiple distances yielding atomic-scale information on the structure and interactions of proteins. Remarkably, by extending the use of REDOR to the determination of ^1^H–X dipolar couplings in recent years, the upper limit of measurable intra- and intermolecular distances has reached ~15–20 Å. As illustrated by the methodological overview and the survey of applications provided in the review, REDOR and REDOR-based solid-state NMR methodologies represent an attractive method for the study of membrane proteins, protein–ligand interactions, and oligomeric assemblies. All these features facilitate the elucidation of the mechanism of transmembrane signaling, enzyme catalysis, viral entry, amyloid formation, and drug binding.
